# Supplementary material for: Can National Registries Contribute to Predict the Risk of Cancer? The Cancer Risk Assessment Model (CRAM)
Source: Cancers (Basel). 2022 Aug 6;14(15):3823. doi: 10.3390/cancers14153823 (PMC9367495; doi:10.3390/cancers14153823)
Supplement: Supplementary file 1 [file cancers-14-03823-s001.zip › cancers-1825457-supplementary.pdf]

Table S1: List of GP´s procedures and measurements and selected specialists

GP´s procedures and measurements

- Consultation
- Telephone consultation
- E-mail consultation
- Blood sample
- Biological material
- Urine dipstick analysis
- Point-of-care Leukocyte/differential count
- Point-of-care C-reactive protein test
- Point-of-care Erythrocyte sedimentation rate
- Point-of-care haemoglobin
- Spirometry
- Spirometry with reversibility
- Peakflow measurement

Selected specialists

- Radiology
- Dermatology
- Rheumatology
- Gynaecological-obstetrical
- Internal medicine
- Surgery
- Neurosurgery
- Neurology
- Ophthalmologist
- Otorhinolaryngology
- Orthopaedic surgery
- Plastic surgery
- Psychiatry
- Paediatrics
- Paediatric psychiatry
- Tropical medicine

Table S2 Coding details on marital status, ethnicity, country of origin, income, educational and occupational status, and comorbidity

- Marital status was categorised into “married/living with someone”, “living alone”.
- Ethnicity was categorised into “Danish”, “immigrant”, “descendant”, and “unknown”.
- Country of origin was categorised into “western countries“, “not-western countries”, and “unknown or missing”
- Income was categorised into “low”, “medium”, “high”
- Occupational status was categorised into “employed”, “unemployed or on welfare payment”, “education”, “early retirement”, “retirement pension”, and “unknown or missing”.

Persons were categorised as living with someone if more than one person aged 18 years or above were living in the same household.

Income and occupational status data were extracted from the Income Statistics Register. We used disposable income after tax payment year 2016. Income was categorised into tertiles, stratified by age groups with five years intervals, which divided an ordered distribution into three parts each containing a third of the population per five years age groups.

Education level was extracted from the Danish Education Register. We used data on highest completed education in year 2017. Level of education was categorised according to ISCED 2011 (UNESCO. International Standard Classification of Education (ISCED) as “low” (ISCED 0-2), “medium” (ISCED 3-4), or “high” education (ISCED 5-8).

Quan´s updated version of the Charlson comorbidity index (CCI) was used to quantify burden of disease classifying comorbid conditions among the respondents (21).We used ICD-10 codes (primary or secondary diagnosis) from NPR for contacts in the period 2007 to 2016, to calculate CCI.

Table S3: Model B; Predictive risk factors including socioeconomics in the development cohort with cancer as outcome: women

Variables	OR (95% CI)	P-value
**Age categories**		
Age 20-29	Ref	
Age 30-34	2.17 (1.67-2.83)	<0.001
Age 35-39	3.00 (2.36-3.83)	<0.001
Age 40-44	4.61 (3.71-5.75)	<0.001
Age 45-49	6.74 (5.47-8.31)	<0.001
Age 50-54	9.07 (7.41-11.10)	<0.001
Age 55-59	12.43 (10.18-15.18)	<0.001
Age 60-64	15.90 (13.02-19.41)	<0.001
Age 65-69	18.28 (14.61-22.88)	<0.001
Age 70-74	19.84 (15.70-25.08)	<0.001
Age 75-79	21.52 (16.95-27.31)	<0.001
Age 80-84	24.61 (19.32-31.36)	<0.001
Age 85-89	19.53 (15.15-25.19)	<0.001
Age 90-94	13.97 (10.42-18.72)	<0.001
Age 95-99	9.33 (5.81-14.97)	<0.001
Age +100	9.27 (2.91-29.58)	<0.001
**ICD-10 codes**		
C44 (skin cancer, other type)	1.48 (1.29-1.69)	<0.001
D05 (Carcinoma in situ of breast)	2.04 (1.52-2.73)	<0.001
D12 (Benign neoplasm of colon, rectum, anus and anal canal)	1.30 (1.16-1.45)	<0.001
D22 (Melanocytic naevi)	1.51 (1.16-1.96)	0.002
D24 (Benign neoplasm of breast)	1.36 (1.13-1.65)	0.001
D25 (Leiomyoma of uterus)	1.26 (1.07-1.49)	0.006
E04 (Other nontoxic goitre)	1.29 (1.12-1.48)	<0.001
E64 (Sequelae of malnutrition and other nutritional deficiencies)	1.77 (1.18-2.67)	0.006
F03 (Unspecified dementia)	0.47 (0.31-0.72)	<0.001
G30 (Alzheimer disease)	0.38 (0.22-0.68)	0.001
G40 (Epilepsy)	1.32 (1.07-1.62)	0.009
I73 (Other peripheral vascular diseases)	1.35 (1.17-1.57)	<0.001
J44 (Other chronic obstructive pulmonary disease)	1.41 (1.28-1.56)	<0.001
J90 (Pleural effusion, not elsewhere classified)	1.66 (1.17-2.36)	0.004
K13 (Other diseases of lip and oral mucosa)	1.70 (1.22-2.37)	0.002
K70 (Alcoholic liver disease)	1.67 (1.19-2.34)	0.003
K83 (Other diseases of biliary tract)	1.62 (1.18-2.23)	0.003
M15 (Polyarthrosis)	0.63 (0.47-0.85)	0.002
N60 (Benign mammary dysplasia)	1.40 (1.16-1.69)	<0.001
O28 (Abnormal findings on antenatal screening of mother)	2.06 (1.29-3.29)	0.003

R00 (Abnormalities of heart beat)	1.24 (1.06-1.46)	0.009
T26 (Burn and corrosion confined to eye and adnexa)	2.47 (1.45-4.21)	0.001
VRK (Perioperativ bleeding (ml))	0.75 (0.61-0.91)	0.004
F22 (Persistent delusional disorders)	0.32 (0.14-0.71)	0.005
**ATC codes**		
A06 (drugs for constipation)	0.81 (0.73-0.90)	<0.001
C08 (calcium channel blockers)	1.08 (1.02-1.14)	0.008
L04 (immunosuppressants)	1.20 (1.05-1.37)	0.006
M05 (drugs for treatment of bone diseases)	0.89 (0.82-0.96)	0.002
N02 (analgesics)	1.07 (1.02-1.13)	0.004
N07 (other nervous system drugs)	1.37 (1.24-1.51)	<0.001
**Practicing specialists (Year(s) before cancer diagnoses)**		
Pediatric psychiatry (3 years)	45.18 (6.12-333.68)	<0.001
Internal medicine (4 years)	0.87 (0.80-0.95)	0.002
Plastic-surgery (1 year)	1.24 (1.06-1.45)	0.007
Psychiatry (5 years)	0.82 (0.72-0.94)	0.004
**GP consultations and procedures (Year(s) before cancer diagnoses)**		
GP consultation (2 years)	0.99 (0.98-0.99)	<0.001
GP consultation (1 year)	1.03 (1.02-1.03)	<0.001
GP e-mail consultation (1 year)	0.99 (0.98-1.00)	0.005
GP haemoglobin (6 years)	0.96 (0.93-0.99)	0.007
GP haemoglobin (1 year)	1.04 (1.01-1.06)	0.002
GP Spirometry (4 years)	1.13 (1.07-1.20)	<0.001
**SES**		
Country, not western	0.66 (0.58-0.76)	<0.001
Occupation, early retirement	1.19 (1.09-1.30)	<0.001
Occupation, retirement	1.27 (1.11-1.45)	0.001
**Constant (baseline odds)**	0.0005949 (0.0004939-0.0007166)	<0.001

Table S4 Model B: Predictive risk factors including socioeconomics in the development cohort with cancer as outcome: men

Variables	OR (95% CI)	P-values
**Age categories**		
Age 20-29	Ref	
Age 30-34	1.71 (1.21-2.42)	0.002
Age 35-39	2.64 (1.94-3.59)	<0.001
Age 40-44	3.43 (2.58-4.55)	<0.001
Age 45-49	5.75 (4.44-7.46)	<0.001
Age 50-54	10.21 (8.00-13.03)	<0.001
Age 55-59	18.33 (14.44-23.25)	<0.001
Age 60-64	28.40 (22.43-35.96)	<0.001
Age 65-69	42.07 (33.32-53.10)	<0.001
Age 70-74	56.47 (44.75-71.26)	<0.001
Age 75-79	57.89 (45.72-73.30)	<0.001
Age 80-84	62.19 (48.87-79.14)	<0.001
Age 85-89	62.95 (48.89-81.04)	<0.001
Age 90-94	52.30 (38.66-70.75)	<0.001
Age 95-99	48.86 (29.02-82.26)	<0.001
Age +100	74.30 (17.86-309.14)	<0.001
**ICD-10 codes**		
B18 (Chronic viral hepatitis)	2.23 (1.64-3.02)	<0.001
D12 (Benign neoplasm of colon, rectum, anus and anal canal)	1.31 (1.20-1.44)	<0.001
E04 (Other nontoxic goitre)	1.47 (1.15-1.87)	0.002
F10 (Mental and behavioural disorders due to use of alcohol)	1.46 (1.30-1.64)	<0.001
F17 (Mental and behavioural disorders due to use of tobacco)	1.34 (1.15-1.57)	<0.001
I70 (Atherosclerosis)	1.27 (1.09-1.47)	0.002
K57 (Diverticular disease of intestine)	0.84 (0.75-0.95)	0.004
K70 (Alcoholic liver disease)	1.57 (1.23-2.01)	<0.001
K83 (Other diseases of biliary tract)	1.78 (1.29-2.46)	<0.001
R29 (Other symptoms and signs involving the nervous and musculoskeletal systems)	0.81 (0.70-0.93)	0.002
R79 (Other abnormal findings of blood chemistry)	1.66 (1.45-1.90)	<0.001
R91 (Abnormal findings on diagnostic imaging of lung)	1.33 (1.16-1.52)	<0.001
T18 (Foreign body in alimentary tract)	1.43 (1.09-1.87)	0.009
T23 (Burn and corrosion of wrist and hand)	1.76 (1.19-2.60)	0.005
T81 (Complications of procedures, not elsewhere classified)	1.19 (1.04-1.36)	0.009
H25 (Senile cataract)	1.20 (1.05-1.36)	0.006
F00 (Dementia in Alzheimer disease)	0.39 (0.23-0.68)	0.001
**ATC codes**		
A12 (mineral supplements)	0.89 (0.82-0.96)	0.004
C08 (calcium channel blockers)	1.11 (1.05-1.16)	<0.001

J01 (antibacterials for systemic use)	1.07 (1.02-1.13)	0.007
N07 (other nervous system drugs)	1.18 (1.07-1.30)	0.001
R01 (nasal preparations)	0.87 (0.81-0.94)	<0.001
R03 (adrenergics, inhalants)	1.11 (1.04-1.19)	0.001
**Practicing specialists (Year(s) before cancer diagnoses)**		
Dermatologist (3 years)	1.05 (1.02-1.07)	<0.001
Paediatric psychiatry (8 years)	1.26 (1.08-1.46)	0.003
Radiology (3 years)	0.91 (0.85-0.97)	0.005
Surgery (1 year)	1.18 (1.07-1.29)	0.001
**GP consultations and procedures (Year(s) before cancer diagnoses)**		
GP consultation (1 year)	1.01 (1.00-1.01)	0.006
GP e-mail consultation (1 year)	0.98 (0.97-0.99)	<0.001
GP blood sample (1 year)	1.03 (1.02-1.05)	<0.001
GP Laboratory test (10 years)	1.06 (1.02-1.10)	0.002
GP C-reactive Protein testing (1 year)	1.03 (1.01-1.06)	0.002
GP Peakflow (9 years)	0.78 (0.66-0.94)	0.008
GP Spirometry (6 years)	1.10 (1.03-1.17)	0.004
GP Spirometry (2 years)	1.09 (1.03-1.16)	0.004
GP Spirometry (5 years)	1.20 (1.05-1.38)	0.010
GP Urine examination (4 years)	0.94 (0.92-0.97)	<0.001
GP Urine examination (1 year)	1.07 (1.04-1.09)	<0.001
Out of hours services, consultation (6 years)	0.90 (0.84-0.97)	0.007
Out of hours services, telephone consultation (2 years)	0.95 (0.91-0.98)	0.005
**SES**		
Countries, western	0.79 (0.69-0.91)	0.001
Countries, not-western	0.60 (0.52-0.69)	<0.001
Income, middle tertile	0.91 (0.86-0.96)	0.001
Income, high tertile	0.86 (0.81-0.91)	<0.001
Occupation, early retirement	1.21 (1.05-1.38)	0.006
Occupation, retirement	1.14 (1.04-1.26)	0.007
**Constant (baseline odds)**	0.0004114 (0.0003266-0.0005182)	<0.001

Table S5: Diagnostic performance of CRAM prediction models for absolute risk cutoffs of 1% and 5%.

Sex	Model	Cohort	Cut off	Probability 0	Probability 1	Sensitivity	Specificity	PPV	NPV
Men	Model A	Validation	1%	0.0022 (0.0020-0.0023)	0.0214 (0.0207-0.0221)	0.7984	0.7157	0.0214	0.9978
Women	Model A	Validation	1%	0.0036 (0.0035-0.0038)	0.0164 (0.0158-0.0170)	0.6618	0.6992	0.0164	0.9964
Men	Model A	Validation	5%	0.0076 (0.0074-0.0078)	0.0375 (0.0305-0.0456)	0.0236	0.9953	0.0375	0.9924
Women	Model A	Validation	5%	0.0075 (0.0073-0.0077)	0.0238 (0.0119-0.0421)	0.0027	0.9992	0.0238	0.9925
Men	Model B	Validation	1%	0.0022 (0.0021-0.0024)	0.0216 (0.0209-0.0224)	0.7935	0.7206	0.0216	0.9978
Women	Model B	Validation	1%	0.0037 (0.0035-0.0039)	0.0165 (0.0159-0.0171)	0.6546	0.7043	0.0165	0.9963
Men	Model B	Validation	5%	0.0076 (0.0073-0.0078)	0.0381 (0.0310-0.0462)	0.0239	0.9953	0.0381	0.9924
Women	Model B	Validation	5%	0.0075 (0.0073-0.0077)	0.0227 (0.0110-0.0414)	0.0025	0.9992	0.0227	0.9925
Men	Model A	Development	1%	0.0022 (0.0021-0.0023)	0.0217 (0.0212-0.0222)	0.8008	0.7147	0.0217	0.9978
Women	Model A	Development	1%	0.0033 (0.0032-0.0035)	0.0167 (0.0163-0.0172)	0.6859	0.6997	0.0167	0.9967
Men	Model A	Development	5%	0.0076 (0.0075-0.0078)	0.0545 (0.0484-0.0611)	0.0333	0.9954	0.0545	0.9924
Women	Model A	Development	5%	0.0074 (0.0072-0.0075)	0.0445 (0.0324-0.0595)	0.0054	0.9991	0.0445	0.9926
Men	Model B	Development	1%	0.0022 (0.0021-0.0023)	0.0220 (0.0215-0.0226)	0.7972	0.7201	0.0220	0.9978
Women	Model B	Development	1%	0.0033 (0.0032-0.0035)	0.0169 (0.0165-0.0174)	0.6826	0.7044	0.0169	0.9967
Men	Model B	Development	5%	0.0076 (0.0075-0.0078)	0.0519 (0.0460-0.0584)	0.0320	0.9954	0.0519	0.9924
Women	Model B	Development	5%	0.0074 (0.0072-0.0075)	0.0451 (0.0327-0.0605)	0.0053	0.9992	0.0451	0.9926

Model A contains ICD-10 codes, ATC codes, and GP and specialist contacts; Model B contains ICD-10 codes, ATC codes, GP and specialist contacts, and socioeconomic status (civil status, income, education level, occupation, and country of origin); PPV, positive predictive value; NPV, negative predicted value.
